# Preventing Depression in Final Year Secondary Students: School-Based Randomized Controlled Trial

**DOI:** 10.2196/jmir.8241

**Published:** 2017-11-02

**Authors:** Yael Perry, Aliza Werner-Seidler, Alison Calear, Andrew Mackinnon, Catherine King, Jan Scott, Sally Merry, Theresa Fleming, Karolina Stasiak, Helen Christensen, Philip J Batterham

**Affiliations:** ^1^ Black Dog Institute University of New South Wales Randwick Australia; ^2^ National Institute for Mental Health Research The Australian National University Canberra Australia; ^3^ Institute of Neuroscience University of Newcastle Newcastle upon Tyne United Kingdom; ^4^ Department of Psychological Medicine University of Auckland Grafton New Zealand

**Keywords:** prevention, depression, adolescent, digital cognitive behavior therapy

## Abstract

**Background:**

Depression often emerges for the first time during adolescence. There is accumulating evidence that universal depression prevention programs may have the capacity to reduce the impact of depression when delivered in the school environment.

**Objective:**

This trial investigated the effectiveness of SPARX-R, a gamified online cognitive behavior therapy intervention for the prevention of depression relative to an attention-matched control intervention delivered to students prior to facing a significant stressor—final secondary school exams. It was hypothesized that delivering a prevention intervention in advance of a stressor would reduce depressive symptoms relative to the control group.

**Methods:**

A cluster randomized controlled trial was conducted in 10 government schools in Sydney, Australia. Participants were 540 final year secondary students (mean 16.7 [SD 0.51] years), and clusters at the school level were randomly allocated to SPARX-R or the control intervention. Interventions were delivered weekly in 7 modules, each taking approximately 20 to 30 minutes to complete. The primary outcome was symptoms of depression as measured by the Major Depression Inventory. Intention-to-treat analyses were performed.

**Results:**

Compared to controls, participants in the SPARX-R condition (n=242) showed significantly reduced depression symptoms relative to the control (n=298) at post-intervention (Cohen *d*=0.29) and 6 months post-baseline (*d*=0.21) but not at 18 months post-baseline (*d*=0.33).

**Conclusions:**

This is the first trial to demonstrate a preventive effect on depressive symptoms prior to a significant and universal stressor in adolescents. It demonstrates that an online intervention delivered in advance of a stressful experience can reduce the impact of such an event on the potential development or exacerbation of depression.

**Trial Registration:**

Australian New Zealand Clinical Trials Registry ACTRN12614000316606; https://www.anzctr.org.au/Trial/Registration/TrialReview.aspx?id=365986 (Archived by WebCite at http://www.webcitation.org/ 6u7ou1aI9)

## Introduction

Depression carries the greatest burden of disease in young people, with an annual prevalence in adolescence of 8% and approximately 80% of these cases experiencing a moderate to severe impact on functioning [1]. It has been estimated that, at most, only 36% of the burden of depression could potentially be alleviated using current knowledge and therapies [2]. Prevention represents an important alternative pathway to reducing this burden, with evidence suggesting that preventive efforts may result in a further 21% to 22% reduction [3]. A number of recent reviews and meta-analyses have found small but consistent effects for the prevention of depression in young people [4]. These reviews indicate mixed findings regarding the relative superiority of universal prevention programs (which target all individuals in a given population regardless of risk) compared to targeted programs (which are directed at high-risk individuals on the basis of the presence of existing risk factors or subthreshold symptoms). However, universal programs may offer additional advantages as they may be easier to implement, are less stigmatizing, and provide greater scope and catchment of individuals who may be on the trajectory toward developing depression but do not yet display symptoms.

The incidence of depression increases monotonically throughout adolescence [5]. However, stressful life events are precursors of and possible causal factors for the onset of depression [6]. Final school examinations are a significant stressor for most adolescents, with exams and academic outcomes endorsed most strongly among school-related stressors [7]. In the Australian context, these final exams determine tertiary education entrance scores, rendering their outcome critically important. More than 40% of final year secondary students report elevated symptoms of depression, anxiety, and stress [8]. In extreme cases, exam stress has been linked to suicidal ideation, behavior, and completion [9]. To date, the potential for prevention has not been examined in the context of school-based stressors. Schools are an ideal location in which to deliver mental health interventions that address these difficulties, not only because young people spend more time in these institutions than any other, but also because of the integral role schools play in students’ social, academic, cognitive, emotional, and behavioral development. While school-based prevention programs have typically taken the format of face-to-face, group-based interventions, online interventions in the school setting are becoming increasingly appealing [10]. Automated online programs guarantee fidelity, since the information cannot be distorted, diluted, or contaminated during delivery [11]. Consequently, implementation of an online universal depression prevention program in the context of a stressor widely experienced in the target sample represented a unique opportunity to evaluate the effectiveness of such an approach.

Our cluster randomized controlled trial (RCT) was designed to test the effectiveness of SPARX-R, an online, cognitive behavioral gamified intervention, in preventing the development or exacerbation of depressive symptoms in final year secondary students relative to an online active control program (lifeSTYLE). SPARX was originally designed as a treatment intervention for depression; however, it was subsequently adapted for use as a universal preventive intervention, known as SPARX-R. SPARX has been shown to result in a decline in symptoms of depression and improved remission rates in a help-seeking sample of adolescents and was found to be non-inferior to treatment as usual (consisting primarily of face-to-face therapy with a counselor, general practitioner, or clinical psychologist) [12]. However, in a recent school trial for individuals with subthreshold depression, SPARX failed to demonstrate effects superior to a control condition despite having a positive impact on depressive symptoms [13]. The authors suggest that the absence of an intervention effect may have been due to the effectiveness of the control condition, which essentially operated as a self-monitoring intervention and may have been sufficient to improve those with mild depressive symptoms. The effectiveness of self-monitoring has been demonstrated in other trials (see, for example, Kauer et al [14]). In our trial, it was predicted that students assigned to the SPARX-R condition would report lower levels of depressive symptoms immediately following the intervention and at 6-month follow-up relative to participants in the attention control condition.

As well as evaluating the impact of this novel intervention on depressive symptoms, this study also examined the effect of SPARX-R on secondary outcomes including anxiety, stigma, academic performance, and suicidal ideation. Given the substantial comorbidity of anxiety and depression and the fact that anxiety symptoms can decline in the context of some depression prevention programs [15], it was expected that participants in the SPARX-R group would also experience reduced anxiety. There is evidence that increased knowledge regarding depression is associated with reduced stigma [16]. Therefore, it was predicted that stigma would also be decreased in the SPARX-R group. Outcomes of final examinations for participants were sought from schools to evaluate whether any impact of the intervention flowed through to academic performance, an expectation arising from the association of depression with poor academic outcomes [17]. Although depression and suicidality are associated [18], they may not follow the same trajectory and so the examination of outcomes pertaining to suicidal ideation and behavior was exploratory.

## Methods

### Study Design

A cluster RCT was conducted with 2 parallel arms consisting of an experimental condition (SPARX-R) and an attention-matched control condition (lifeSTYLE). The trial was designed for implementation in schools in accordance with the Australian academic calendar: baseline assessments were conducted at the start of the academic year (February), followed by a 5-week intervention phase and post-intervention assessments. Six-month follow-up assessments were conducted midyear, just prior to a significant period of academic assessments that contribute to students’ final secondary school grade (the exam period). The final follow-up assessment was conducted 18 months after baseline. At this time, participants had graduated from high school, and the majority were engaged in post-secondary studies. This trial received ethics approval from the New South Wales Department of Education and Training (SERAP2014001), the University of New South Wales (HC14105), and the Australian National University (2014/325). The study protocol is published [19].

### Participants

The trial was conducted in selective and partially selective government secondary schools in metropolitan Sydney. Selective schools use an entry exam to enroll students with superior academic ability, while partially selective schools offer both selective and comprehensive (non-selective) streams. Academically gifted students were selected because final school exam outcomes are critical to them. They are particularly sensitive to the stressful nature of exams, with studies finding they experience adverse stress reactions to academic failures coupled with pressure to succeed from parents, schools, the media, and students themselves [20]. The academic workload and considerable number of hours spent studying in a demanding educational program is likely to compound this pressure and act as an additional stressor [21]. These attributes made this group a particularly relevant sample in which to test the stress-diathesis model of depression.

All adolescents enrolled in their final year of secondary school in participating schools were invited to participate in the trial. All students at participating partially selective schools were eligible to participate in the trial; however, streaming status (ie, selective or comprehensive) was recorded and evaluated in analyses. Due to the universal nature of the study, there were no exclusion criteria. Written, informed consent was sought from students and their parents prior to the start of the trial. Students who did not provide consent were able to access the assigned intervention; however, only those with consent completed the research questionnaires.

### Interventions

#### SPARX-R

SPARX-R is a revised version of SPARX, which was developed as an unguided, interactive program using the format of a fantasy game providing cognitive behavioral skills to treat mild to moderate symptoms of depression in help-seeking adolescents.

SPARX-R provides users with the same skills as those in SPARX [12]; however, the revised version is framed in preventive terms. For example, participants are told that “this version of SPARX was made to help young people who are having hassles and feeling down, stressed, or angry a lot of the time. Even if you are doing fine, SPARX-R can help strengthen your skills for dealing with problems when they do come along.” Further, for the version of SPARX-R used, care was taken to ensure that terminology and local helplines and services were suitable for Australia. SPARX-R users choose and personalize an avatar and are led through the program by a virtual guide who provides context and links the content of the program to their real-life experiences. The user navigates their way through a series of challenges within a fantasy world that has been overrun by GNATs (gloomy, negative, automatic thoughts), with the mission of restoring balance in the game world. The program has 7 modules (levels), each of which takes approximately 20 to 30 minutes to complete. Participating schools scheduled curriculum time for participants to complete 1 to 2 modules per week, allowing a few days in between modules for students to process what they learned and practice new skills before beginning the next module. The intervention was completed over the course of 5 to 7 weeks in class under teacher supervision. The modules cover the following topics: finding hope, being active, dealing with strong emotions, overcoming problems, recognizing unhelpful thoughts, challenging unhelpful thoughts, and bringing it all together. Key skills taught by the program were relaxation, activity scheduling and behavioral activation, emotion regulation, interpersonal skills, problem solving, cognitive restructuring, and distress tolerance [12]. SPARX-R was delivered to students on desktop computers via the Internet in school classrooms and supplemented with a paper notebook for students to review key messages from each module and record personal comments. A trailer for how SPARX works can be found at sparx.org.nz.

#### lifeSTYLE

lifeSTYLE is an adaptation of an interactive, online program originally developed as a control intervention for a trial targeting adults with suicidal thoughts [22]. The format and structure of the program was retained, but the content was adapted to suit adolescents. This active comparison was used to control for the large placebo effect commonly seen in depression prevention and treatment trials, which has been cited as a key methodological weakness in many studies. The aim of the intervention was to provide an engaging and useful resource for young people that matched the intervention in terms of duration and attention without providing any direct mental health content. As with SPARX, lifeSTYLE consisted of 7 modules, each of which took 20 to 30 minutes to complete. The program covers the following topics: independence, participating in your community, work skills, mobile phone safety and hygiene, healthy skin, sustainable eating, and maintaining a healthy home environment. Each module includes information about the specified topic as well as interactive activities such as quizzes, mythbusters, videos, and scenarios to which students can respond. As with SPARX-R, the intervention was delivered online to students in school classrooms.

### Randomization and Masking

Each participating school (cluster) was randomized to SPARX-R or lifeSTYLE by a statistician not involved in the implementation of the trial and blind to the identity of the schools. All study staff remained blind to condition except for the 2 trial managers involved in the communication of the materials and instructions to the schools (YP, AWS). The method of Carter and Hood [23] sought to ensure balance between arms. Balance variables were gender, number of enrolled students, Index of Community Socio-Educational Advantage for each school, and language background other than English.

No research personnel were directly involved in the delivery of the prevention interventions. Schools were not informed whether their assigned program represented the experimental or control condition. All outcome measures were self-report and were completed privately via an online portal. The trial statistician was blind to school allocation.

### Procedure

Following school-level randomization, participants completed baseline assessments and their allocated intervention modules. Program sessions were scheduled at each school’s convenience. For the most part, students collectively completed the sessions during the first 20 to 30 minutes of a designated class period and then resumed their regular lessons for the remainder of the period. The majority of participants completed their post-intervention assessments in scheduled class sessions within 1 week of the intervention period. A follow-up assessment session was scheduled approximately 6 months after the baseline assessment (July; approximately 2 weeks prior to the exam period). A final follow-up assessment was scheduled 18 months after the baseline assessment; approximately 7 to 8 months after participants had completed their final exams. Exam results were provided by school administrators approximately 6 weeks after the final exam period ended (November-December).

### Outcome Measures

#### Primary Outcome Measure

The Major Depression Inventory (MDI) is a 12-item self-report measure of depressive symptoms [24]. The items of the MDI evaluate the presence and duration of depressive symptoms according to both *International Statistical Classification of Diseases and Related Health Problems, 10th Revision*, (ICD-10) and *Diagnostic and Statistical Manual of Mental Disorders, 4th Edition*, (DSM-IV) criteria. Respondents rate the degree to which they have experienced each of 10 symptoms over the preceding 2 weeks on a 6-point scale, ranging from 0 (at no time) to 5 (all of the time). The MDI has the following cut-off points: 21 to 25 for mild depression, 26 to 30 for moderate depression, and 31 to 50 for severe depression [25]. The MDI has acceptable sensitivity and specificity for the diagnosis of depression according to the ICD-10 and DSM-IV [24]. In this trial, Cronbach alpha, assessed at baseline, was .88.

#### Secondary Outcome Measures

The Spence Children’s Anxiety Scale (SCAS) is a 44-item measure comprising 6 subscales. The scale was designed to measure the severity of children and adolescents’ anxiety symptoms based broadly on DSM-IV criteria for anxiety disorders [26]. Respondents rate the degree to which they experience each symptom on a 4-point frequency scale, ranging from 0 (never) to 3 (always). Only items on the social phobia and generalized anxiety subscales were administered. The scale has demonstrated high reliability [26]. In this trial, Cronbach alphas for the social phobia and generalized anxiety scales were .76 and .81, respectively.

Three items from the Youth Risk Behavior Survey concerning suicidal thoughts, plans, and attempts over the previous month were used to assess suicidality. Endorsement of any of these items triggered the trial risk management protocol. Studies have shown that the suicidality items demonstrate reliability [27].

The Depression Stigma Scale (DSS) is an 18-item measure that assesses personal and perceived stigma toward depression. In this study, only the 9-item personal stigma subscale was used, as this subscale was most relevant. Items require the participant to rate how strongly they agree with a statement about depression (eg, “people with depression are unpredictable”) on a 5-point Likert scale ranging from 0 (strongly disagree) to 4 (strongly agree). The sum of each of the items yields a total stigma score, where higher scores indicate greater stigma. For this sample, Cronbach alpha was .77.

Final exam results were also collected and standardized using the Australian Tertiary Admission Rank such that the 2 groups could be compared on academic outcomes.

### Sample Size

Based on previous depression prevention research using an online intervention [15], a post-intervention Cohen *d* effect size for the primary outcome measure of .20 was used for sample size calculations. Power was set at 80%, alpha=.05 (2-tailed), and a correlation of .5 assumed between baseline and endpoint scores. To allow for possible clustering effects, a design effect was calculated assuming an intraclass correlation (ICC) of .02 and an average class size of 25. The estimate of the ICC was derived from a previous Australian school-based study that found a nonsignificant ICC of .02 [15]. The estimated sample size was 1166. The total target size sample size was set at 1600, 800 students per condition, in order to accommodate possible attrition at a rate up to 20% [28].

The protocol [19] specified 2 stages of recruitment over consecutive years, with a planned analysis conducted using data from the first cohort as soon as available. In addition to evaluating effectiveness of the intervention, a review of first cohort outcomes was planned, aimed at identifying any refinement of content and mode of delivery required before proceeding to the second stage of recruitment. Schools recruited to the first cohort had an enrollment of 1677 eligible students. Under the assumptions outlined above, for this cohort alone, there was 80% power to detect an effect size of 0.40 standard deviations between groups.

### Data Analysis

Primary analyses were undertaken on an intent-to-treat (ITT) basis. Effectiveness of SPARX-R was established using a planned contrast of change from baseline to post-intervention in the active compared to placebo condition on the MDI using a mixed-model repeated measures (MMRM) analysis that incorporates all available data under the missing-at-random assumption [29]. School was included in analyses as a random effect to evaluate and accommodate clustering effects. Variables used in determining allocation balance were evaluated and retained in 2 sensitivity analyses, 1 adjusting for characteristics differing at baseline and 1 excluding non-selective students. An unconstrained variance-covariance matrix was used to model within-individual dependencies, and degrees of freedom were estimated using Satterthwaite’s method [30]. Between-group effect sizes (Cohen *d*) were calculated using observed mean loss/gain scores from pre-intervention to post-intervention, from pre-intervention to 6-month follow-up, and from pre-intervention to 18-month follow-up, divided by pooled standard deviation at the later time-point. Positive effect sizes indicate changes in favor of the intervention condition.

Although our MMRM analytic approach was conducted under the robust assumption that data were missing-at-random, we also conducted a completer analysis, excluding participants who did not complete a follow-up assessment. This analysis was conducted to provide evidence for whether findings were consistent across alternative assumptions about missingness. Due to high attrition from the trial at follow-up, consistency across multiple analytical approaches may provide added confidence in the findings. Outcomes of the completer analysis are briefly summarized with comparison to the ITT analyses, as completer analyses may be more prone to bias [31].

Prevalence rates of major depressive disorder and suicidal ideation between the 2 treatment arms at post-intervention, 6 months, and 18 months were compared using Fisher exact test. Analyses of secondary variables also used MMRM methods. Subsidiary analyses of students who completed post-test evaluations (protocol compliers) were used to estimate the efficacy of SPARX-R in participants who completed sufficient modules from the program to have a potential clinical impact (estimated to be 4 or more modules) [12]. Separate analyses for completer subgroups (eg, completed 0 to 3 modules vs 4 to 7 modules) were also undertaken, and clinically significant change (improvement and decline) was assessed in program completers. Due to scheduling difficulties, 2 participants in lifeSTYLE and 22 in SPARX-R completed a 6-month assessment after completion of exams. As the stressor of interest had consequently passed, the 6-month data from these participants were excluded from analyses. All analyses were undertaken using SPSS version 23 (IBM Corp). The trial was registered with the Australian New Zealand Clinical Trials Registry [ANZCTRN12614000316606].

## Results

**Figure 1:**
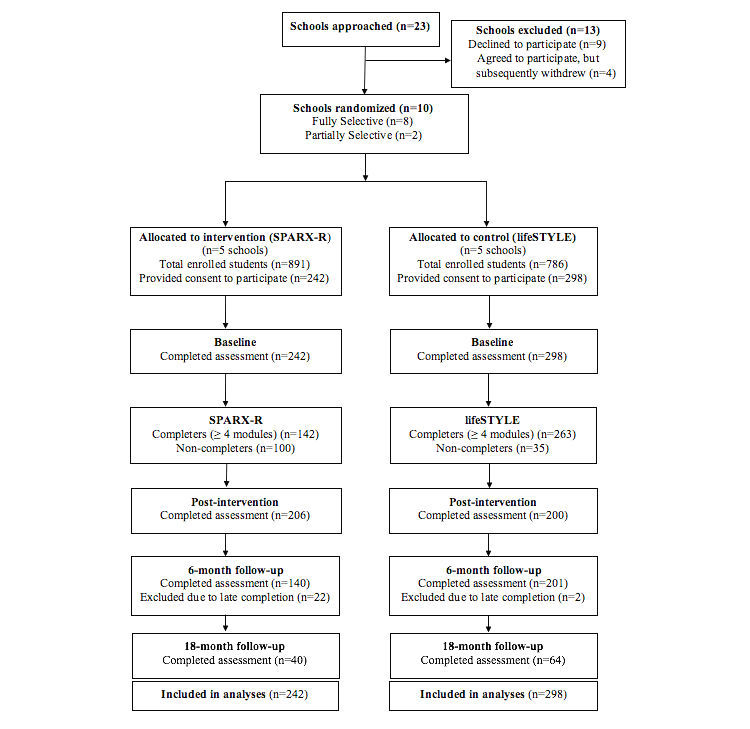
CONSORT Diagram.

A total of 23 secondary schools were approached between August 1 and November 28, 2014, to participate in the trial. Of 14 schools who initially agreed to participate, 4 dropped out prior to randomization leaving 10 schools contributing 7 to 126 participants each. A total of 540 students provided personal consent and obtained parental consent to participate. The Consolidated Standards of Reporting Trials (CONSORT) flowchart outlines recruitment, randomization, and participation (see Figure 1). Baseline assessments commenced in February 2015 with 6-month follow-up assessments completed by August 2015 and 18-month follow-up assessments completed by August 2016.

Sample characteristics are shown in Table 1. There were no differences between the SPARX-R and lifeSTYLE conditions on the basis of country of birth, language primarily spoken at home, age, depression symptoms, generalized anxiety disorder symptoms, social phobia symptoms, or subjective health. However, males, students from selective programs, and students living with both parents together had significantly greater representation in the SPARX-R condition relative to lifeSTYLE. Participants in SPARX-R completed fewer intervention modules than those in lifeSTYLE. Estimated marginal means and standard errors were derived from models fitted for primary and secondary continuous outcomes at each time point by condition (see Table 2).

**Table 1:** Sample characteristics for the SPARX-R and lifeSTYLE conditions.

Characteristic		SPARX-R n=242 n (%)	lifeSTYLE n=298 n (%)	Chi-square	*P* value
**School program**					
	Non-selective	4 (1.7)	72 (24.2)	55.9	<.001
	Selective	238 (98.3)	226 (75.8)		
**Sex**					
	Male	117 (48.3)	82 (27.5)	24.9	<.001
	Female	125 (51.7)	216 (72.5)		
**Country of birth**					
	Australia	195 (80.6)	236 (79.2)	0.2	.69
	Other	47 (19.4)	62 (20.8)		
**Language spoken at home**					
	English	132 (54.5)	175 (58.7)	1.0	.33
	Other	110 (45.4)	123 (41.3)		
**Live with both parents together**					
	Yes	216 (89.3)	235 (78.9)	10.5	<.001
	No	26 (10.7)	63 (21.1)		

**Table 2:** Mean and standard error for continuous outcomes at each time point (estimated from mixed effect models).

Test	SPARX-R				lifeSTYLE			
	Baseline n=242 mean (SE)	Post n=206 mean (SE)	6 months n=140 mean (SE)	18 months n=40 mean (SE)	Baseline n=298 mean (SE)	Post n=200 mean (SE)	6 months n=201 mean (SE)	18 months n=64 mean (SE)
MDI^a^	14.9 (0.9)	11.9 (0.9)	13.3 (1.0)	10.0 (1.1)	14.4 (0.9)	14.7 (0.9)	15.3 (0.9)	11.9 (1.0)
SCAS GAD^b^	6.7 (0.4)	5.9 (0.4)	6.5 (0.6)	5.1 (0.5)	6.8 (0.4)	6.4 (0.4)	6.6 (0.4)	5.7 (0.5)
SCAS SA^c^	7.2 (0.5)	6.4 (0.5)	6.5 (0.5)	6.1 (0.6)	7.4 (0.4)	6.8 (0.5)	6.6 (0.5)	6.5 (0.5)
DSS^d^	9.4 (1.0)	8.9 (1.1)	8.4 (1.1)	8.1 (1.1)	8.8 (1.0)	8.8 (1.0)	8.1 (1.0)	7.8 (1.0)

^a^MDI: Major Depression Inventory.

^b^SCAS GAD: Spence Child Anxiety Scale–Generalized Anxiety Disorder.

^c^SCAS SA: Spence Child Anxiety Scale–Social Anxiety.

^d^DSS: Depression Stigma Scale.

The ICC for depressive symptoms was 0.017. Primary analyses showed that participants in the SPARX-R condition had a significantly greater reduction in MDI scores than those in the lifeSTYLE condition both post-intervention (*t*_(416.1)_=−4.78, *P*<.001) and at 6-month follow-up (*t*_(317.6)_=−2.76, *P*=.01). The effect was not significant at 18-month follow-up (*t*_(132.1)_=−1.82, *P*=.07). Effect sizes were small post-intervention (Cohen *d*=0.29, 95% CI 0.09 to 0.49), at 6-month follow-up (Cohen *d*=0.21, 95% CI −0.01 to 0.42), and 18-month follow-up (Cohen *d*=0.33, 95% CI −0.06 to 0.73).

When completer analyses were conducted, the effects of the intervention on depression scores remained significant (*F*_(3, 254.0)_=8.00, *P*<.001), with significant effects at post-test (*t*_(410.4)_=–4.84, *P*<.001) and 6-month follow-up (*t*_(368.1)_=–2.82, *P*=.005) but not at 18 months (*t*_(132.4)_=–1.93, *P*=.055), consistent with the ITT analysis. Secondary outcomes also remained nonsignificant in completer analyses.

For participants who completed 4 or more modules, the intervention was effective at both post-intervention (*t*_(328.3)_=−4.55, *P*<.001) and 6 months (*t*_(313.6)_=−2.68, *P*=.01) but not at 18-month follow-up (*t*_(116.4)_*=* −1.54, *P*>.05). Conversely, those who completed fewer modules showed no significant intervention effect (*P*=.83 post-intervention, *P*=.57 at 6 months) except for at 18 months (*t*_(15.5)_*=* −3.08, *P=*.01). A clinically significant improvement in depression, as indicated by the Reliable Change Index [32], was found in a larger proportion of completers of SPARX-R than controls at post-test (65/206, 31.6%, vs 26/200, 13.0%, *P*<.001) and 6-month follow-up (35/126, 27.8%, vs 33/202, 16.3%, *P*=.02) but not at 18-month follow-up (15/41, 37%, vs 17/64, 27%, *P*=.29).

No significant effects were found on any of the secondary outcomes. Generalized anxiety and social anxiety were significantly reduced at post-intervention relative to baseline for both groups, but there was no significant condition by time interaction effect for either anxiety outcome. There were no differences between conditions in the prevalence of depression at any time, based on MDI caseness criteria [24], with caseness of 4.9% (10/206) in SPARX-R and 8.5% (17/200) in lifeSTYLE at post-intervention (*P*=.17), 11.9% (15/126) and 9.4% (19/202), respectively, at 6 months (*P*=.46), and 2.4% (1/41) and 4.7% (3/64), respectively, at 18 months (*P*>.99). Few participants reported suicidal ideation at any time point, and no significant differences were found between groups: 3.4% (7/205) in SPARX-R and 4.1% (8/196) in lifeSTYLE at post-intervention (*P*=.80), 2.4% (3/126) and 4.0% (8/198), respectively, at 6 months (*P*=.54), and 3% (1/39) and 5% (3/62), respectively, at 18 months (*P*>.99). Notably, academic outcomes did not differ between the 2 groups (*P*=.41).

## Discussion

### Principal Findings

The online gamified prevention intervention, SPARX-R, was effective at reducing depression symptoms in secondary students prior to their final exams. The effect size was small but robust and corresponds to previous results from cognitive behavior therapy (CBT)–based school programs delivered face-to-face based on recent meta-analyses [4]. Effects remained significant after controlling for selective streaming status as well as characteristics found to differ substantially at baseline. Completion of the program was associated with clearly improved primary outcomes, such that SPARX-R completers reported fewer depressive symptoms postintervention and at 6-month follow-up than their lifeSTYLE counterparts, while no intervention effect was detected for noncompleters. Differences in rates of clinically significant change were demonstrated, implying that the intervention produces meaningful change in depressive symptoms under universal administration. Given that a small effect at the population level can have a substantial impact on overall mental health, we assess the impact of the magnitude of effect of the intervention as promising. This is an important finding as it highlights the effectiveness of SPARX-R in the context of a truly universal sample comprising those who are at elevated risk for developing depression and those who are not.

No intervention effects were found for generalized anxiety, a finding at variance with the use of SPARX as a treatment intervention in the initial study [12], although anxiety was not measured in the second SPARX study [13]. The overall reduction in anxiety observed in the course of the trial is somewhat surprising as it might be expected to increase with the approach of final exams.

No between-group differences were found in depression caseness, suicidality, or academic outcomes. Low base rates for caseness and suicidality would likely have obscured any effects that existed. It is unclear why decreased symptoms did not improve academic performance, (although findings were in the expected direction), but it is possible that due to the high achieving nature of the sample there was insufficient variability in academic performance to detect change or students may have already been performing at ceiling. This was also a universal group of relatively psychologically well students, so differences may be more likely to emerge in a clinical sample or at least at the more severe end of the spectrum [17]. This question will need to be addressed in future prevention research.

### Strengths and Limitations

A key strength of the study is its implementation in school settings where the intervention will ultimately be delivered, directly supporting the translational capacity of this research into practice. The trial is noteworthy having been mounted in a sample faced with a meaningful, real-world stressor. Indeed, we believe that the anticipation of the stressor may have led to greater engagement, addressing a problem that has been identified in more recent digital trials [33]. Thus, the significant effects detected are of great potential importance, given that they were obtained under pragmatic, real-world conditions. The use of an active attention control condition, which was matched in terms of length, delivery format, and interactivity, means the effects are attributable to the intervention rather than involvement in a research trial or generic placebo effects.

Several limitations associated with this study warrant mention. In common with most Internet-delivered universal interventions, completion rates of the active intervention were modest (59% in the SPARX-R group; defined as completion of 4 or more of 7 modules) although substantially higher than comparable studies in secondary schools [15]. While this rate is lower than the 86% completion rate observed in the SPARX treatment trial [12], it is important to note that this sample was universal rather than help-seeking, which may account for differences in motivation. Completion may have also been compounded by technical difficulties. Several technical problems occurred during implementation (particularly in schools allocated to the SPARX-R intervention), which impacted some students’ ability to complete all of their allocated modules during class time. This was largely due to the excessive load on the school information technology (IT) system associated with multiple students simultaneously accessing the online research platform and downloading the SPARX-R game files. Indeed, the completion rate of 4 or more modules in the lifeSTYLE group (which did not encounter the same degree of difficulty due to a smaller load on IT systems) was substantially higher (88%). In response to these issues, the stability and download size of the SPARX-R program has been reviewed with the assistance of software developers. A mobile app of the intervention is also being developed and will be tested in the future.

Our decision to recruit in academically selective schools means that caution must be exercised in generalizing results to the broader school population. However, approximately 80% of our sample was born outside Australia and over half did not speak English at home, suggesting this was a culturally, if not academically, diverse sample.

Resources and feasible sample size precluded mounting a trial with incidence of clinically diagnosed depressive disorder as its primary outcome. This limitation is shared by nearly all such school-based trials and might be precluded in any case due to the demands of the education curriculum in the last year of high school. Accordingly, this trial aimed to maximize ecological validity in order to determine the true effect of a prevention program under real-world conditions, although we acknowledge that a small proportion of participants may have met criteria for the disorder at baseline.

### Prior Research

To date, the majority of school-based depression prevention trials have delivered material face-to-face in groups. Five published studies have examined the effectiveness of online prevention interventions in the school setting. Calear and colleagues [15] were the first to demonstrate the utility of an online CBT program (MoodGYM) in high school students, demonstrating a positive effect for the program at post-intervention and 6-month follow-up for anxiety (Cohen *d*=0.15 and 0.25, respectively) and depressive symptoms (in males only; *d*=0.43 and 0.31, respectively). Wong and colleagues [34] conducted an RCT investigating 2 separate, blended online/offline CBT interventions (Thiswayup Schools) targeting depression and anxiety compared to regular health classes. Students who received the depression intervention reported lower symptoms of both depression (*d*=0.14) and anxiety (*d*=0.29) at post-intervention. More recently, 2 trials have not shown significant effects, findings that have been interpreted as a failure to engage or attributed to the strength of the control group intervention [13,33]. Our study suggests that the context in which the intervention is offered must also be taken into consideration. Adult findings indicate the power of preparatory CBT in the context of a stressor. For example, in 1 study doctors in training were randomly assigned to 2 study groups, either MoodGYM or an attention control group, prior to taking up their medical internship, a period of high stress [35]. Results showed that suicide ideation was less frequently endorsed in the intervention group than the control over the course of the year. Therefore, interventions delivered prior to major transitions or major stressors may provide greater engagement (both from the students and the school administrators) and be more effective than those delivered at arbitrary times.

SPARX-R can be delivered without direct facilitation and requires only minimal supervision from teachers, reducing the human resources required to deliver face-to-face school-based programs, and may offer an effective and scalable universal prevention approach for students. Indeed, it could be the first step in a stepped care approach to mental health interventions, whereby young people within the school setting are provided with access to a universal, skills-oriented intervention, with those who require additional support being stepped up to more intensive care as needed.

### Conclusion

This is the first trial to demonstrate a preventive effect on depression prior to a significant and universal stressor in adolescents. It extends the evidence base for appropriately developed CBT skills delivered through a gamified program as being broadly effective for young people regardless of whether their symptoms are minimal, elevated, or meet diagnostic criteria. Our results highlight the potential utility of delivering prevention interventions in advance of a key stressor and the program feasibility and effectiveness of using an engaging interactive tool for depression prevention. Major implementation studies of these types of digital interventions are now warranted.

## Abbreviations

ANZCTR: Australian and New Zealand Clinical Trials Registry
CBT: cognitive behavior therapy
CONSORT: Consolidated Standards of Reporting Trials
DSM-IV: Diagnostic and Statistical Manual of Mental Disorders, 4th Edition
DSS: Depression Stigma Scale
GNAT: gloomy, negative, automatic thought
ICC: intraclass correlation
ICD-10: International Statistical Classification of Diseases and Related Health Problems, 10thRevision
IT: information technology
ITT: intention-to-treat
MDI: Major Depression Inventory
MMRM: mixed-model repeated measures
NHMRC: National Health and Medical Research Council
SCAS: Spence Child Anxiety Scale
RCT: randomized controlled trial
